# An Internal Standard-Assisted Synthesis and Degradation Proteomic Approach Reveals the Potential Linkage between VPS4B Depletion and Activation of Fatty Acid **β**-Oxidation in Breast Cancer Cells

**DOI:** 10.1155/2013/291415

**Published:** 2013-02-04

**Authors:** Zhongping Liao, Stefani N. Thomas, Yunhu Wan, H. Helen Lin, David K. Ann, Austin J. Yang

**Affiliations:** ^1^Greenebaum Cancer Center, University of Maryland School of Medicine, Baltimore, MD 21201, USA; ^2^Department of Pharmacology and Molecular Sciences, Johns Hopkins University School of Medicine, Baltimore, MD 21205, USA; ^3^Department of Epidemiology and Public Health, University of Maryland School of Medicine, Baltimore, MD 21201, USA; ^4^Department of Molecular Pharmacology, Beckman Research Institute, City of Hope Medical Center, Duarte, CA 91010, USA; ^5^Department of Anatomy and Neurobiology, University of Maryland School of Medicine, Baltimore, MD 21201, USA

## Abstract

The endosomal/lysosomal system, in particular the endosomal sorting complexes required for transport (ESCRTs), plays an essential role in regulating the trafficking and destination of endocytosed receptors and their associated signaling molecules. Recently, we have shown that dysfunction and down-regulation of vacuolar protein sorting 4B (VPS4B), an ESCRT-III associated protein, under hypoxic conditions can lead to the abnormal accumulation of epidermal growth factor receptor (EGFR) and aberrant EGFR signaling in breast cancer. However, the pathophysiological consequences of VPS4B dysfunction remain largely elusive. In this study, we used an internal standard-assisted synthesis and degradation mass spectrometry (iSDMS) method, which permits the direct measurement of protein synthesis, degradation and protein dynamic expression, to address the effects of VPS4B dysfunction in altering EGF-mediated protein expression. Our initial results indicate that VPS4B down-regulation decreases the expression of many proteins involved in glycolytic pathways, while increased the expression of proteins with roles in mitochondrial fatty acid **β**-oxidation were up-regulated in VPS4B-depleted cells. This observation is also consistent with our previous finding that hypoxia can induce VPS4B down-regulated, suggesting that the adoption of fatty acid **β**-oxidation could potentially serve as an alternative energy source and survival mechanism for breast cancer cells in response to hypoxia-mediated VPS4B dysfunction.

## 1. Introduction

VPS4B, a member of the AAA (ATPases associated with diverse cellular activities) protein family, plays an important role in the lysosomal degradation pathway, which functions in ligand-induced membrane receptor downregulation. In lysosomal degradation, endocytosed receptors are sorted into multivesicular bodies (MVBs), which requires the sequential assembly of endosomal sorting complex required for transport I, II, and III (ESCRT-I, -II, and -III) on the endosomal membrane [1]. VPS4B functions to dissociate ESCRT-III from endosomes for further rounds of endosomal sorting [2]. Expression of functionally inactive VPS4B results in the accumulation of receptors on abnormally enlarged endosomes (class E compartments) and the abolishment of MVB biogenesis [3–6]. As an essential ESCRT-III interacting protein, VPS4B is also involved in protein trafficking among membrane compartments and in signal transduction [4, 5, 7, 8].

Epidermal growth factor- (EGF-) mediated signaling is one of the most important signaling pathways for cell growth, proliferation, invasion, and metastasis in breast cancer [9]. Upon ligand binding, the EGF receptor (EGFR) is phosphorylated, promoting downstream signal transduction, while it is internalized and degraded within the MVB-lysosome [10]. EGFR signaling is initiated at the cell membrane; however, late signaling propagation occurs on the endosomes [11]. We and others have reported that dysfunction of VPS4B not only leads to delayed EGFR degradation, but also to prolonged and altered intracellular EGFR signaling in abnormally enlarged endosomal compartments [4–6, 12]. To further understand the role of VPS4B dysfunction in breast cancer, we set out to determine the consequences of altered EGFR signaling: changes in protein synthesis and degradation, as well as protein dynamics in VPS4B downregulated breast cancer cells. Altered protein synthesis and degradation of cancer-related proteins is involved in cellular transformation and cancer progression [13–18]. Understanding protein synthesis and degradation is essential to fully appreciate cellular dynamics and develop more effective strategies to treat cancer. 

Traditional protein synthesis and degradation studies rely on radiolabel tracer labeling (pulse) followed by exposure to nonradioactive medium (chase). However, these approaches are often designed to address the turnover of a specific protein. Recently, high throughput synthesis and degradation mass spectrometry (SDMS) has become a novel approach to study global protein turnover, in which protein degradation and synthesis can be measured simultaneously and unambiguously with minimal cell perturbation [19]. In this approach, proteins are first metabolically labeled by stable isotope labeling with amino acids in cell culture (SILAC) followed by chasing in normal medium. As a result, the preexisting proteins are labeled with stable isotope amino acids, while the newly synthesized proteins only incorporate regular amino acids. These differential stable isotope-labeled proteins can be discriminated from each other and quantified by mass spectrometry based on the differences in masses of their peptides following enzymatic digestion. Since Pratt and coworkers introduced the use of mass spectrometry in protein synthesis and degradation studies [20]; this approach has been applied to numerous systems, such as *Escherichia coli *[21], *Mycobacterium tuberculosis* [22], *Mycoplasma pneumoniae* [23], *Streptomyces coelicolor *[24], *Saccharomyces cerevisiae *[20], HeLa cells [25, 26], human adenocarcinoma cells [27], chicken skeletal muscle [28, 29], and mice [30, 31]. 

One of the major assumptions of using a pulse-chase labeling experiment to study the dynamics of protein expression is that the level of protein expression is always under the steady state. In SILAC-based protein synthesis and degradation experiments, the degradation rate constant of targeted proteins can be readily derived from the decrease of relative isotope abundance (RIA) or the percentage of preexisting labeled protein [20]. On the contrary, it is more difficult to calculate the rate of protein synthesis in general since proteins are synthesized continuously in cultured proliferating cells, thus the rate of protein accumulation is not likely to be under steady state. In order to bypass such steady state assumption, the incorporation of isobaric tags for relative and absolute quantitation (iTRAQ) [24] or the introduction of an internal control, such as another set of labeled cells [26], permits the simultaneous measurement of protein synthesis, degradation, and expression under dynamic conditions, such as cell proliferation.

Dysfunction of VPS4B leads to EGFR accumulation and prolonged activation [4–6, 12], which could contribute to cell proliferation and growth. To estimate protein synthesis and degradation rates in the context of altered EGFR signaling caused by downregulation of VPS4B, we independently developed an approach called internal standard-assisted synthesis and degradation mass spectrometry (iSDMS) that normalizes protein abundance across different samples and time points and permits the comparison of protein synthesis, degradation, and expression measurements. A similar approach has also been developed recently by Boisvert et al. to study global protein turnover in HeLa cells [26]. Through this iSDMS analysis, we compared the synthesis and degradation rates of more than 700 proteins between VPS4B downregulated SKBR3 cells and the parental SKBR3 cells. We found that VPS4B downregulation resulted in differential protein expression in energy metabolism pathways by altering the synthesis and degradation of related proteins, in which glycolysis proteins were downregulated, while mitochondrial fatty acid *β*-oxidation proteins were upregulated. The adoption of fatty acid *β*-oxidation as an alternative energy source could be an unrevealed survival mechanism for breast cancer cells with VPS4B dysfunction. 

## 2. Materials and Methods

### 2.1. Cell Culture

SKBR3 cells were obtained from the American Type Culture Collection (Rockville, MD). VPS4B knock-down SKBR3 (SKBR3_shVPS4B) cells were generated by transducing SKBR3 cells with a lentivirus harboring shRNA against VPS4B (SA Biosciences/Qiagen, Frederick, MD) as previously described [32]. To study the role of VPS4B downregulation on the dynamics of protein expression by iSDMS, SKBR3_shVPS4B, and the parental SKBR3 cells were cultured in DMEM SILAC medium (Pierce/Thermo Scientific) supplemented with 28 mg/L ^13^C_6_-arginine (Arg6, purity 97–99%, Cambridge Isotope Laboratories), 72 mg/L D_4_-lysine (Lys4, purity 96–98%, Cambridge Isotope Laboratories), 10% dialyzed fetal bovine serum (FBS, Invitrogen), and 1% antibiotic antimycotic solution (Invitrogen). For SKBR3_shVPS4B cells, 2 *μ*g/mL puromycin were added to the culture medium to maintain the selection pressure. After five passages, cells were starved in serum free SILAC medium for 18 hr. Following three washes with Dulbecco's Phosphate-buffered Saline (DPBS, Invitrogen), cells were stimulated with 100 ng/mL EGF and subsequently “chased” for 0, 2, 6, 12, and 24 hr in the presence of DMEM “light” SILAC medium (Pierce/Thermo Scientific) supplemented with light arginine (Arg0), lysine (Lys0) (Sigma), 10% dialyzed FBS, and 1% antibiotic antimycotic solution. At the end of each chase time period, cell pellets were collected and frozen in liquid nitrogen and stored in −80°C until analysis. To prepare the “heavy-” labeled internal standard, SKBR3_shVPS4B cells were metabolically labeled with ^13^C_6_^15^N_4_-arginine (Arg10) and ^13^C_6_^15^N_2_-lysine (Lys8) by culturing in DMEM “heavy” SILAC medium supplemented with 28 mg/L ^13^C_6_^15^N_4_-arginine (Arg10, purity 97–99%, Cambridge Isotope Laboratories), 72 mg/L ^13^C_6_^15^N_4_-lysine (Lys8, purity 97–99%, Cambridge Isotope Laboratories), 10% dialyzed FBS, and 1% antibiotic antimycotic solution (Invitrogen). The incorporation of Arg10 and Lys8 in the internal standard SKBR3_shVPS4B cells was ~98% as determined by mass spectrometric analysis of tryptic peptides isolated from the Arg10 and Lys8 labeled SKBR3_shVPS4B cells. Cells were harvested and stored as described above.

### 2.2. Preparation of Cell Lysates and Enzymatic Digestion

Cell pellets were lysed and sonicated in 4% SDS,100 mM Tris-HCl (pH 7.6) lysis buffer containing 5 U/mL benzonase nuclease (Novagen) and complete EDTA-free protease inhibitor cocktail (Roche). After removal of cell debris by centrifugation at 10,000 ×g at room temperature for 10 min, protein concentration was measured in triplicate using the BCA protein assay kit (Thermo Scientific/Pierce). 180 *μ*g of protein from each time point were mixed with 60 *μ*g of protein internal standard labeled with ^13^C_6_^15^N_4_-arginine10 and ^13^C_6_^15^N_2_-lysine8. Dithiothreitol (DTT, Sigma) and Tris (2-carboxyethyl) phosphine (TCEP, Thermo Scientific/Pierce) were added to the mixture to final concentrations of 100 mM and 10 mM, respectively. Mixtures were kept at 90°C for 10 min. After cooling to room temperature, the cell lysates were processed by the Filter Aided Sample Preparation (FASP) procedure [33] using 30 k VIVACON 500 filtration units (Sartorius Biolab) with modifications. Briefly, cell lysates were first reduced by mixing with 200 *μ*L of UA buffer (8 M urea in 100 mM Tris-HCl pH 8.5) containing 5 mM TCEP, loaded into the filtration units, and centrifuged at 14,000 ×g for 15 min. At the end of the reduction reaction, the samples were washed three times in 200 *μ*L of UA buffer followed by centrifugation at 14,000 ×g for 15 min. Alkylation was performed by adding 100 *μ*L of 50 mM iodoacetamide in UA buffer and incubating at room temperature for 30 min. Excess iodoacetamide was eliminated by centrifugation, followed by three washes with 200 *μ*L of UB buffer (8 M urea in 100 mM Tris-HCl, pH 8.0).

At the end of reductive alkylation reaction, samples were digested by sequential addition of proteases Lys-C and trypsin in the filtration units. Briefly, 2 *μ*g of endoproteinase Lys-C (Roche) in 40 *μ*L water were first added to the filtration units and kept at room temperature in a humidified chamber overnight for 16 hr. After Lys-C digestion, samples were diluted with 200 *μ*L of 50 mM NH_4_HCO_3_, and 4 *μ*g of trypsin (Promega) were added to the reaction mixture and incubated at 37°C for another 6–8 hr. After digestion, the peptides were collected by centrifugation of the filtration units, followed by two washes with 50 *μ*L of 50 mM NH_4_HCO_3_. All peptides containing filtrates were pooled and acidified by the addition of trifluoroacetic acid (TFA, Sigma) to a final concentration of 1%. Acidified peptides were desalted by a Sep-Pak C18 cartridge (Waters) and dried by SpeedVac (Thermo Scientific). Dried peptides were stored in a −80°C freezer until analysis. 

### 2.3. Peptide Fractionation

Peptides were fractionated using stop and go extraction (STAGE) tips made in house with strong anion exchange (SAX) disks as described in [34–36]. The STAGE tips were assembled by stacking six layers of Empore anion exchange membrane disks (3 M) into a 200 *μ*L micropipet tip. Two stacked tips were used to separate 100 *μ*g of peptides. Britton and Robinson buffer (BR buffer), composed of 20 mM acetic acid, 20 mM phosphoric acid, and 20 mM boric acid and titrated with NaOH to the desired pH, was used in peptide separation. The tip was wet with methanol and washed with 1 M NaOH, followed by equilibration with 100 *μ*L of BR buffer pH 11. Peptides were loaded at pH 11, and fractions were subsequently eluted with BR buffer of pH 11, 8, 6, 5, 4, and 3, respectively. The flow through and all the fractions were acidified by adding TFA to a final concentration of 1% and desalted by a STAGE tip containing three layers of C18 membrane disks (3 M). Peptides were then dried and stored as mentioned above.

### 2.4. LC-MS/MS Analysis

Peptides were dissolved in 0.5% acetic acid (solvent A) and separated on a 10 cm reverse-phase PicoFrit spray tip (New Objective, Woburn, MA) packed in house with sub-2 *μ*m C18 resin (Prospereon Life Science, IL), using a nanoflow Xtreme simple liquid chromatography system (Microtech/CVC) coupled to a hybrid linear ion trap-Orbitrap mass spectrometer (LTQ Orbitrap, Thermo Scientific). Peptides were loaded onto the column with solvent A at a flow rate of 0.6 *μ*L/min and eluted with a 180 min linear gradient at a flow rate of 0.2 *μ*L/min. A gradient of 2–60% solvent B (40% acetonitrile, 0.5% acetic acid) was applied to the SAX flow through, fractions eluted with pH 11 and pH 8 buffer, a 2–65% solvent B gradient was used for the pH 6 and pH 5 fractions, and a 5–70% solvent B gradient was used for the pH 4 and pH 3 fractions. After the linear gradient, the column was washed with 95% solvent B and reequilibrated with 95% solvent A. Seven fractions from one sample were run sequentially followed by a 40 min wash with 80% acetonitrile with 0.5% acetic acid.

Mass spectra were acquired in the positive ion mode applying a data-dependent automatic switch between the survey scan and MS/MS acquisition. The survey MS1 scans were acquired in the Orbitrap using a mass range of *m/z* 400–1,600 at a resolution of 60,000 at 400 *m/z*. The MS1 target value was 100,000. MS/MS scans were acquired in the linear ion trap on the 5 most intense ions in each survey scan with dynamic exclusion of previously selected ions; repeat count 1 and exclusion duration 15 seconds. The fragmentation was performed by collision-induced dissociation (normalized collision energy 35%) with a target value of 30,000. Ion selection threshold was 5,000 counts. Charge state screening was enabled, and +1 ions were excluded from fragmentation. Other mass spectrometric parameters that were spray voltage 1.35 kV; no sheath and auxiliary gas flow; ion transfer tube temperature 180°C; activation *q* = 0.25; activation time of 30 ms were applied in MS2 acquisitions.

### 2.5. Peptide Identification

Data were searched against a concatenated forward/reverse database, which was built based on a UniProt human database (downloaded on Oct. 18, 2010), using SEQUEST (Bioworks 3.3.1 SP1, Thermo Scientific). Enzyme specificity was set to trypsin and allowed two missed cleavages. Database search parameters were precursor peptide mass tolerance 50 ppm and fragment ion tolerance 1 amu. Carbamidomethyl cysteine was set as a fixed modification, and oxidized methionine was set as a variable modification. Additional variable modifications included arginine +6.02013, arginine +10.00827, lysine +4.02510, and lysine +8.01420. The peptides were initially filtered by the following criteria: *X*_corr_ ≥ 2.5, 3.0, and 3.5 for 2+, 3+, and 4+ peptides, respectively, and ΔCn > 0.1. The false discovery rate (FDR) was calculated by dividing the number of false positive peptides identified in the reverse database by the number of total identified peptides [37]. In this study, the FDR was 0.94%.

### 2.6. Calculation of Peptide Relative Abundance

By adding the internal standard, three populations of a given peptide were present in the mixture: the peptide containing regular arginine (Arg0) or lysine (Lys0) (light-labeled peptide), the peptide containing ^13^C_6_-arginine (Arg6) or D_4_-lysine (Lys4) (medium-labeled peptide), and the peptide containing ^13^C_6_^15^N_4_-arginine (Arg10) or ^13^C_6_^15^N_2_-lysine (Lys8) (heavy-labeled internal standard peptide).

The relative abundance of light peptide (*A*_*l*_) was defined as the ratio of the peak intensities of unlabeled peptide (*I*_*l*_) to the intensities of internal standard peptide (*I*_*h*_), (1). Similarly, the relative abundance of labeled peptide (*A*_*m*_) was defined as the ratio of the peak intensities of labeled peptide (*I*_*m*_) to *I*_*h*_, (2). An in-house SILAC-based mass spectrometry quantitation software, IsoQuant (http://www.proteomeumb.org/MZw.html) [38], was used to automatically integrate the isotopic peak intensities of each peptide and calculate *A*_*l*_ and *A*_*m*_. The relative abundance of total peptide (*A*_tot_) was calculated by summing the relative abundance of light unlabeled peptide (*A*_*l*_) and labeled peptide (*A*_*m*_), (3)

(1) $$\begin{array}{c} A_{l}=\frac{I_{l}}{I_{h}}, \end{array}$$

(2) $$\begin{array}{c} A_{m}=\;\frac{I_{m}}{I_{h}}, \end{array}$$

(3) $$\begin{array}{c} A_{\text{tot}}=A_{l}+A_{m}. \end{array}$$

### 2.7. Calculation of Peptide Degradation Rate Constant

Since the SILAC labeled peptides were only subjected to degradation, the degradation rate constant could be derived from the changes in relative abundance of labeled peptides. Since protein degradation rate follows first-order kinetics [39], the relative abundances of the preexisting labeled peptides over time (*A*_*m*_*t*_) were fit to exponential decay curves to derive the degradation rate constant (*λ*). At least four time points were required to find the best fitting curve. Only *A*_*m*_*t*_ and *t* that were significantly correlated (*P* < 0.05) with a coefficient of determination (*R* ^2^) greater than 0.8 were selected to derive the degradation rate constant from the exponential decay equation as follows:

(4) $$\begin{array}{c} A_{m\_t}=a\times e^{-\lambda t}, \end{array}$$

where *a* was the corrected-normalized initial peptide amount.

### 2.8. Calculation of Peptide Synthesis Rate

Peptide synthesis rate was defined as the rate of change of the relative abundance of the newly synthesized peptide over time. The relative abundance of the newly synthesized peptide can be calculated by the relative abundance of the unlabeled peptide directly, if the basal label efficiency reaches 100%. However, due to different cell types, cell culture conditions, and the purity of the stable isotope-labeled amino acids, the basal labeling efficiency was not 100%, which consequently resulted in the detection of unlabeled peptides at the beginning of the chase period. In this study, at the beginning of the chase period, ~10% and 30% of the peptides were unlabeled in SKBR3_shVPS4B and SKBR3 cells, respectively. To calculate the relative abundance of the newly synthesized peptide, the relative abundance of the unlabeled peptide has to be corrected by subtracting the relative abundance of the preexisting unlabeled peptide at 0 hr, as well as the later time points. Since both the preexisting unlabeled and labeled peptides were subjected to degradation, the preexisting unlabeled peptide at each time point (*A*_pre_*l*_*t*_) was calculated by

(5) $$\begin{array}{c} A_{\text{pre}\_l\_t}=A_{l\_0}\times e^{-\lambda t}, \end{array}$$

where *A*_*l*_0_ was the relative abundance of the unlabeled peptide at 0 hr. 

The relative abundance of the newly synthesized peptide at each time point (*A*_*l*_*t*_′) was calculated by

(6) $$\begin{array}{c} A_{l\_t}^{\prime }=A_{l\_t}-A_{\text{pre}\_l\_t}, \end{array}$$

where *A*_*l*_*t*_ was the abundance of the total unlabeled peptide at each time point.

Protein synthesis follows zero-order kinetics [39]. The relative abundances of newly synthesized peptides over time *A*_*l*_*t*_′ were fit to linear curves to derive the synthesis rates (*γ*). At least four time points were required to find the best fitting curve. Only *A*_*l*_*t*_′ and *t* that were significantly correlated (*P* < 0.05) with a *R* ^2^ greater than 0.8 were selected to derive the synthesis rate from the following linear equation:

(7) $$\begin{array}{c} A_{l\_t}^{\prime }=\gamma \times t+b, \end{array}$$

where *b* was the corrected initial peptide amount.

### 2.9. Protein Synthesis Rate, Degradation Rate Constant, and Relative Abundance

The protein synthesis rate, degradation rate constant, and relative protein abundance were calculated by the mean of their identified peptides' synthesis rates, degradation rate constants, and total relative peptide abundance.

## 3. Results

### 3.1. iSDMS Analysis of Dynamic Protein Profiles

**Figure 1:**
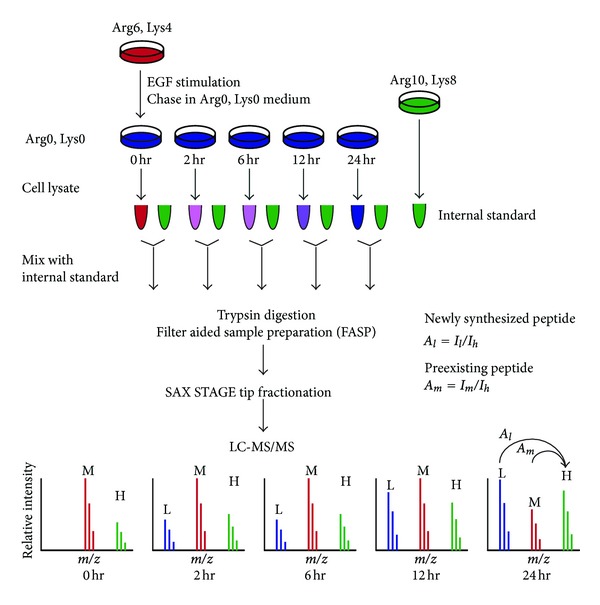
Using internal standard-assisted synthesis and degradation mass spectrometry (iSDMS) to study the roles of EGF on global protein synthesis and degradation. SKBR3_shVPS4B and the parental SKBR3 cells were first labeled with ^13^C_6_-arginine (Arg6) and D_4_-lysine (Lys4) medium (labeled in red). After overnight serum starvation, Arg6/Lys4-labeled cells were stimulated with 100 ng/mL EGF in medium supplemented with regular arginine (Arg0) and lysine (Lys0) for 0, 2, 6, 12, and 24 hr (labeled in blue). Protein isolated from SKBR3_shVPS4B cells labeled with ^13^C_6_^15^N_4_-arginine (Arg10) and ^13^C_6_^15^N_2_-lysine (Lys8)—internal standard (labeled in green)—was spiked into each sample at a ratio of 1 : 3 (wt/wt). The mixtures were digested by the Filter Aided Sample Preparation (FASP) procedure, followed by strong anion exchange (SAX) peptide fractionation. Peptides were analyzed by online LC-MS/MS using an LTQ-Orbitrap mass spectrometer. The relative abundance of the newly synthesized (*A*_*l*_) or preexisting peptides (*A*_*m*_) was defined as the ratio of mass spectrometric peak intensities of the unlabeled peptides (*I*_*l*_) or Arg6/Lys4-labeled peptides (*I*_*m*_) to the intensities of the Arg10/Lys8-labeled peptides (*I*_*h*_), respectively.

 Our earlier report has shown that hypoxia leads to the abnormal accumulation of EGFR and subsequent alteration of cell signaling in breast cancer. To further understand the role of VPS4B dysfunction in global protein dynamics upon EGF treatment, we decided to measure the rates of protein synthesis and degradation in VPS4B downregulated SKBR3 (SKBR3_shVPS4B) and the parental SKBR3 cells using an internal standard assisted synthesis and degradation mass spectrometry (iSDMS) approach. As shown in Figure 1, both cultured SKBR3_shVPS4B and SKBR3 cells were first metabolically labeled with stable isotopic amino acids arginine, and lysine (Arg6/Lys4, labeled in red), and then chased in medium containing regular arginine and lysine amino acids (Arg0/Lys0, labeled in blue) in the presence of EGF. As a result, one can monitor the rates of protein degradation simply by measuring the decrease of Arg6/Lys4-labeled proteins, while newly synthesized proteins can only be monitored by the incorporation of Arg0 and Lys0 amino acids. Since the depletion of VPS4B expression is likely going to have some effects on global protein expression, it is necessary to normalize the steady state protein profiling and differential gene expression between VPS4B ablation and the parental cells. To overcome the issue of differential gene expression between the two cell lines, an internal standard-cell lysates metabolically labeled with Arg10/Lys8 (labeled in green) was added to each time point in order to normalize the relative abundance of Arg6/Lys4 or Arg10/Lys6-labeled peptides between SKBR3_shVPS4B and SKBR3 cells. The detailed calculation of peptide relative abundance is described in the Section 2.

**Figure 2:**
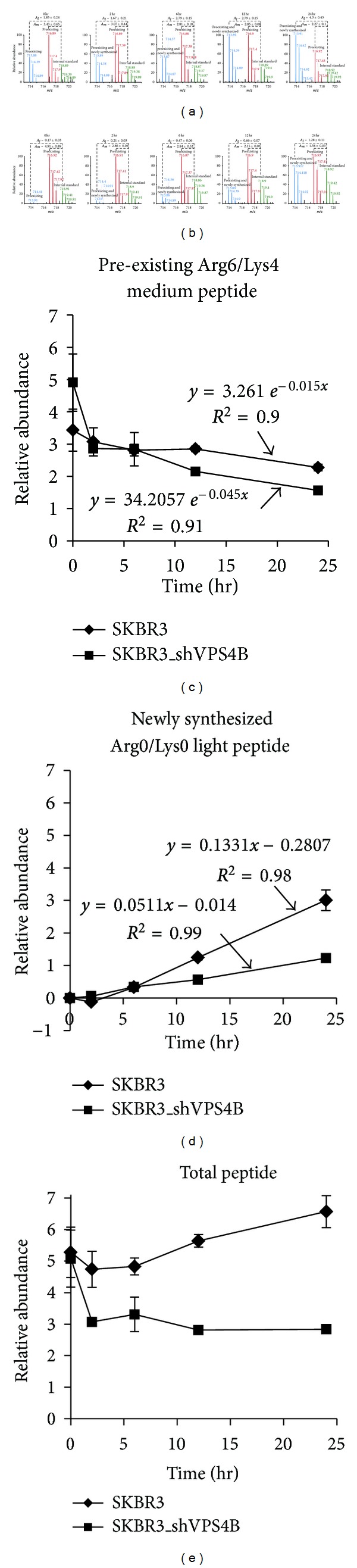
VPS4B downregulation decreases the expression of fatty acid synthase (FASN) in SKBR3_shVPS4B cells. Representative MS1 spectra of fatty acid synthase peptide SLLVNPEGPTLMR in SKBR3 (a) and SKBR3_shVPS4B cells (b). The relative abundance of unlabeled peptides (*A*_*l*_, labeled in blue) or labeled peptides (*A*_*m*_, label in red), expressed as mean ± standard  deviation, was calculated by our in-house software, IsoQuant [38]. VPS4B downregulation increased the degradation rate of the SLLVNPEGPTLMR FASN peptide (c) and decreased its synthesis rate (d) in SKBR3 cells, which was related to the decrease of its relative abundance after EGF treatment (e).

Figures 2(a) and 2(b) are representative MS spectra of tryptic peptides, SLLVNPEGPTLMR, derived from chain A of human fatty acid synthase (FASN) in SKBR3 (Figure 2(a)) and SKBR3_shVPS4B cells (Figure 2(b)) identified in the chase time periods of 0, 2, 6, 12, and 24 hr. The decreasing abundance of the preexisting Arg6/Lys4-labeled peptides (labeled in red) between the 12 and 24 hr chase periods in both cell lines clearly indicates that the degradation of this peptide can be monitored by this type of approach. To determine the role of VPS4B on protein turnover, a first-order exponential decay curve fitting was constructed (see Section 2.7 for more details). As indicated in Figure 2(c), the peptide derived from fatty acid synthase (SLLVNPEGPTLMR) clearly had a faster degradation rate in SKBR3_shVPS4B cells (labeled in squares, degradation rate constant = 0.015 h^−1^, *R* ^2^ = 0.91) than SKBR3 cells (labeled in diamonds, degradation rate constant = 0.045 h^−1^, *R* ^2^ = 0.90).

On the other hand, the calculation of synthesis rate is much more difficult because the pool of unlabeled Arg0/Lys0 (labeled in blue) peptides consisted of both newly synthesized peptides during the chase period, and preexisting unlabeled peptides, which were present at the beginning of the experiment due to incomplete labeling of Arg6/Lys4 amino acids. Since preexisting unlabeled (Arg0/Lys0) peptides were being degraded during the course of the experiment, it is necessary to normalize the relative abundance of the remaining (nondegraded) preexisting unlabeled peptides at each time point based on their degradation rate constant(see Section 2.8 for more details).  Figure 2(d) is a representative time-course analysis of newly synthesized human FASN peptides (SLLVNPEGPTLMR). This result suggests that the FASN peptide (SLLVNPEGPTLMR) had a slower synthesis rate in SKBR3_shVPS4B cells (labeled in squares, synthesis rate = 0.055 h^−1^, *R* ^2^ = 0.99) as compared to SKBR3 cells (labeled in diamonds, synthesis rate = 0.133 h^−1^, *R* ^2^ = 0.98).

Because iSDMS allows one to measure the rates of protein synthesis and degradation simultaneously, it is therefore possible to determine the dynamics of protein expression in VPS4B-depleted cells in response to EGF.  Figure 2(e) is a representative time course analysis of FASN dynamic expression. In this study, the relative abundance of a given peptide (*A*_tot_) of FASN (SLLVNPEGPTLMR) from each time point was calculated and normalized by summing the relative abundance of unlabeled peptide (*A*_*l*_) and labeled peptide (*A*_*m*_) ((3), Section 2). It is clear that the relative abundance of FASN expression in SKBR3_shVPS4B cells (Figure 2(e), labeled in squares) was drastically reduced after two hours of EGF treatment. Together with the result from the degradation study of FASN in Figure 2(c), this observation suggests that the decrease of FASN expression is largely due to the increased turnover rate of FASN in SKBR3_shVPS4B cells (Figure 2(c), labeled in squares). Interestingly, after 6 hr of EGF treatment the rate of FAN degradation was very similar between SKBR3_shVPS4B and SKBR3 cells, indicating that the sudden increase of FASN turnover rate in SKBR3_shVPS4B cells could be potentially regulated by EGF-related cell signaling pathways.

### 3.2. Changes of the Dynamic Proteome Profile in Relationship to VPS4B Depletion

VPS4B is essential for the formation of MVB and has also been documented to play a pivotal role in regulating the degradation of various membrane receptors. Therefore, we decided to examine the consequence of VPS4B ablation on the dynamic proteome profile using our SKBR3_shVPS4B model system. Briefly, both cultured SKBR3_shVPS4B and the parental control SBKR3 cells grown on SILAC “medium,” Arg6/Lys4, medium were treated with 100 ng/mL of EGF and chase labeled in SILAC “light,” Arg0/Lys0, medium for 2, 6, 12, and 24 hr as described in the Section 2. At the end of each chase period, SKBR3_shVPS4B and SKBR3 cells were collected and analyzed by a FASP-based SAX fractionation and LC-MS/MS analysis using a hybrid Orbitrap mass spectrometer. MS/MS raw files were then subjected to database search and quantification analysis using our in-house software IsoQuant [38]. However, in order to compare the degradation rate constants and synthesis rates of each peptide in SKBR3_shVPS4B and SKBR3 cells at the proteome level, we required that the “same” peptides should be identified and quantified at each time point in both cell lines throughout the entire course of the experiment in all five time points. As indicated in Figures 2(c)-2(d), the relative peptide abundance and the time period used to calculate both degradation rate constants and synthesis rates also need to be highly correlated (*P* < 0.05; *R* ^2^ > 0.8) with at least four time points in order to derive the rates. These criteria have significantly reduced the depth of proteome; however, we were able to generate a highly statistically relevant dataset on the effects of VPS4B in modulating proteome dynamics. Overall, we have identified more than 15,000 total unique peptides assigned to more than 4,500 protein groups in both SKBR3_shVPS4B and SKBR3 cells. Over 70% of the proteins were assigned with a minimum of two peptides. We obtained the degradation rate constants, synthesis rates, and total peptide profiles of 1623 peptides and 723 proteins. At the global level, the overall mean protein synthesis rate in SKBR3_shVPS4B cells was 0.058 ± 0.010 h^−1^, which was similar to that of the SKBR3 cells, 0.057 ± 0.012 h^−1^(see Supplemental Figures 1(a) and 1(b) available online at http://dx.doi.org/10.1155/2013/291415). This result suggests that VPS4B does not affect global protein synthesis in general in response to EGF treatment. Similarly, VPS4B depletion also does not have any systematic effect on global protein degradation, because the mean protein degradation rate constant was 0.033 ± 0.012 h^−1^ (*t*_1/2_ = 20 hr) in SKBR3_shVPS4B cells, which was only slightly higher than that of the SKBR3 cells, 0.028 ± 0.010 h^−1^ (*t*_1/2_ = 24 hr) (Supplemental Figures 1(c) and 1(d)).

Because the heavy SILAC Arg10/Lys8-labeled internal protein mixture was spiked into every time point, it is possible to analyze the effect of VPS4B downregulation on global protein expression. To address the effect of VPS4B downregulation on dynamic protein profiles, we defined increased and decreased protein synthesis, degradation, and relative protein abundance in SKBR3_shVPS4B cells as those values greater than 1.5-fold that of the SKBR3 cells (SKBR3_shVPS4B versus SKBR3 ratio <0.67 or >1.5). Among 723 proteins, the majority did not show changes in their rates of protein synthesis (80.9% of proteins), degradation (77.2% of proteins), or relative protein abundance (90.2% of proteins) (Supplemental Table 1). As expected, downregulation of VPS4B has a rather limited effect on the alteration of overall protein synthesis, because only ~10% of proteins displayed changes in their rates of protein synthesis. On the other hand, ~20% of proteins had increased rates of degradation in SKBR3_shVPS4B cells, suggesting that VPS4B depletion potentially affects and enhances the turnover of certain proteins. At the global level, our data suggest that VPS4B downregulation does not change the dynamic protein profiles of most proteins in SKBR3 cells. Despite the VPS4B-depleted cell line having a rather different growth phenotype and sensitivity to hypoxia as compared to the parental cells, we found that the overall distributions of relative protein abundance at 24 hr in SKBR3 and SKBR3_shVPS4B cells are also very similar (Supplemental Figures 1(e) and 1(f)).

**Figure 3:**
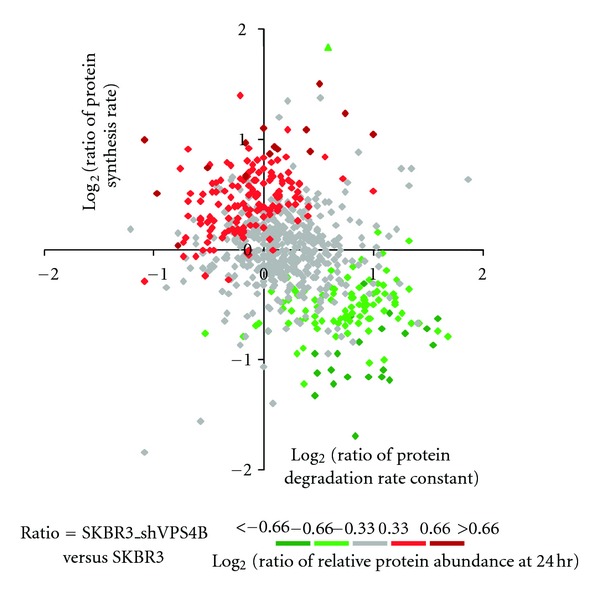
The role of VPS4B downregulation on the alteration of global protein synthesis and degradation after EGF treatment. Ratios of synthesis rates of SKBR3_shVPS4B versus SKBR3 cells (*y* axis) are plotted against ratios of degradation rate constants of SKBR3_shVPS4B versus SKBR3 cells (*x* axis). Diamonds represent log⁡_2_ ratios of protein expression at 24 hr between the two cell lines, and the relative values of which are indicated on the color scale.

In this study, we found that among 33 proteins with increased relative abundance (Supplemental Table 2), 63.6% had increased synthesis only, 9.1% had decreased degradation only, and 9.1% had both (Table 1). Among 38 proteins with decreased relative abundance (Supplemental Table 3), 18.4% had decreased synthesis only, 15.8% had increased degradation only, and 65.8% had both (Table 2). Because the steady state accumulation of proteins is determined by their rates of synthesis and degradation, we therefore decided to further examine the relationship between the steady state protein expression and protein synthesis and degradation in response to the downregulation of VPS4B expression. The relative protein abundance ratios between the two cell lines (SKBR3_shVPS4B versus SKBR3) were plotted against the ratios of protein synthesis rates (Supplemental Figure 2(a)) and the ratios of protein degradation rate constants (Supplemental Figure 2(b)). As indicated in Figure 3, it is clear that the VPS4B-mediated decrease of protein expression is largely due to increased protein degradation, while the increase of VPS4B-mediated protein expression is directly related with increased protein synthesis. For instance, proteins with higher relative abundance in SKBR3_shVPS4B cells (Figure 3, red diamonds) were found to have both lower degradation rate constants and higher synthesis rates (Figure 3, 2nd quadrant). Conversely, those proteins with lower relative abundance have higher degradation rate constants and lower synthesis rates (Figure 3, 4th quadrant, labeled in green diamonds).

### 3.3. VPS4B Downregulation Alters Energy Metabolism in SKBR3 Cells

**Figure 4:**
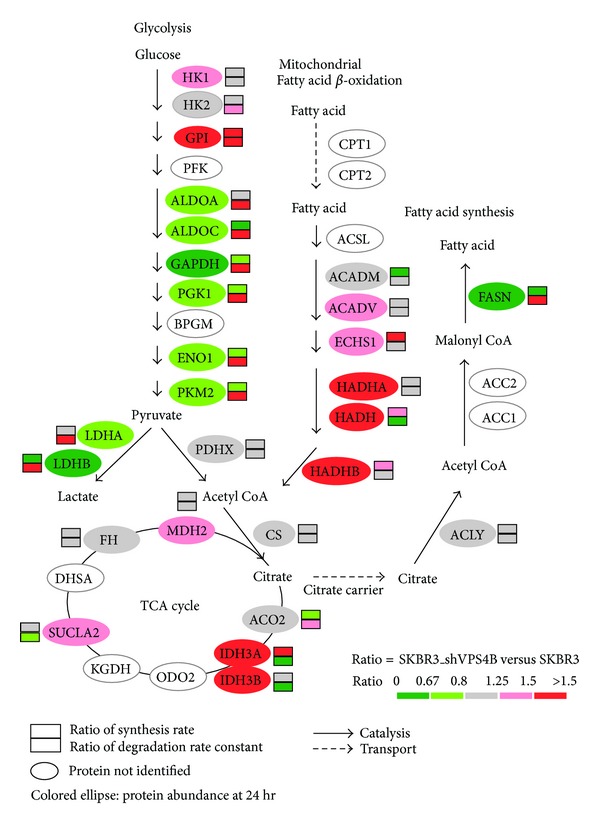
VPS4B downregulation increases the expression of mitochondrial fatty acid *β*-oxidation related proteins and down regulates the expression of glycolysis related proteins in SKBR3 cells. The energy metabolism pathway was adapted from the Kyoto Encyclopedia of Genes and Genomes (KEGG) [40]. Colors represent the ratios of relative protein abundance at 24 hr (shaded ellipses), protein synthesis rate (shaded upper rectangles), and protein degradation rate constant (shaded lower rectangles) in SKBR3_shVPS4B versus SKBR3 cells. Red color represents protein with increased expression and green color represents protein with decreased expression.

To further understand the biological consequences of VPS4B ablation in EGF-mediated cell signaling, a gene ontology and Kyoto Encyclopedia of Genes and Genome (KEGG) pathway analysis was performed [40]. As indicated in Figure 4, proteins involved in energy metabolism, in particular glycolysis and mitochondrial fatty acid *β*-oxidation pathways, are significantly affected in SKBR3_shVPS4B cells upon EGF treatment. In humans, glycolysis and fatty acid *β*-oxidation are the two main sources of acetyl-CoA for the tricarboxylic acid cycle (TCA) to generate adenosine triphosphate (ATP). We found that the expression of many key glycolytic enzymes, such as glyceraldehyde-3-phosphate dehydrogenase (GAPDH) and L-lactate dehydrogenase B chain (LDHB), fructose-bisphosphate aldolase A and C (ALDOA, ALDOC), phosphoglycerate kinase 1 (PGK1), alpha-enolase (ENO1), pyruvate kinase isozymes M1/M2 (PKM2), and L-lactate dehydrogenase A chain (LDHA), was drastically reduced in SKBR3_shVPS4B cells. As indicated in Figure 4, the decreased expression of these proteins is mainly caused by the simultaneously increased degradation and decreased synthesis rates of these proteins as the consequence of VPS4B downregulation.

 Interestingly, the expression of mitochondrial trifunctional protein alpha-subunit (HADHA/ECHA), mitochondrial trifunctional protein beta-subunit (HADHB/ECHB), and mitochondrial hydroxyacyl-coenzyme A dehydrogenase (HADH) was increased more than 1.5-fold in SKBR3_shVPS4B cells. Mitochondrial very long-chain specific acyl-CoA dehydrogenase (ACADV) and mitochondrial enoyl-CoA hydratase (ECHM) were increased more than 1.25-fold. The increased expression of these mitochondrial proteins and proteins involved in fatty acid *β*-oxidation are primarily caused by the increased protein synthesis rate, rather than caused by decreased protein degradation. On the other hand, fatty acid synthase (FASN) expression was decreased in SKBR3_shVPS4B cells with increased degradation and decreased synthesis rates. Taken together, our results suggest that downregulation of VPS4B expression can potentially alter the energy metabolism in breast cancer, suggesting that under either hypoxia or VPS4B depletion, fatty acid tends to be oxidized to generate energy rather than being stored, consequently replacing glucose as a main energy source. 

## 4. Discussion

Dysfunction of VPS4B results in altered endosomal trafficking of membrane receptors, such as EGFR, as well as EGFR-associated signaling molecules [3–8, 12]. Toward gaining a more thorough understanding of the consequences of altered EGFR signaling caused by VPS4B dysfunction, we developed an iSDMS method to identify changes in the protein synthesis, degradation rates, and dynamic protein expression, in breast cancer SKBR3_shVPS4B cells. In this study, we obtained dynamic profiles of more than 700 proteins in both SKBR3_shVPS4B and SKBR3 cells in response to EGF. Most importantly, we have also identified many previously unidentified energy metabolism pathways that were altered as a result of VPS4B downregulation in breast cancer. In particular, we found that downregulation of VPS4B expression has a profoundly negative effect on the expression of several key proteins involved in glycolysis and fatty acid synthesis, suggesting that TCA cycle and ATP energy metabolism could be compromised. In order to overcome this defect in ATP generation, we found that the expression of many proteins involved in fatty acid *β*-oxidation is also elevated in the VPS4B-depleted cells, indicating that the activation of fatty acid *β*-oxidation could serve as a potential survival mechanism and ultimately lead to its resistance to chemotherapy and hypoxia.

Protein degradation rate constants vary among different cell types and tissues. In the same biological system, the degradation rate constants also vary widely among different proteins. In *E. coli*, protein degradation rate constants range from 0.017 h^−1^ (*t*_1/2_ = 40 hr) to 0.058 h^−1^ (*t*_1/2_ = 11.9 hr), with a mean value of 0.03 h^−1^ (*t*_1/2_ = 23 hr) [23]. In human A549 adenocarcinoma cells, the degradation rate constants range from 2 × 10^−5^ (*t*_1/2_ = 69,000 hr) to 5.4 h^−1^ (*t*_1/2_ = 0.13 hr), with a mean of 0.081 h^−1^ (*t*_1/2_ = 8.5 hr) [27]. In our study, we found that among more than 700 proteins, protein degradation rate constants ranged from 0.012 (*t*_1/2_ = 57 hr) to 0.116 h^−1^ (*t*_1/2_ = 5.9 hr) with a mean of 0.033 h^−1^ (*t*_1/2_ = 20 hr) in SKBR3_shVPS4B cells and 0.01 to 0.087 h^−1^ with a mean of 0.028 h^−1^ (*t*_1/2_ = 24 hr) in SKBR3 cells. Due to the sensitive of our current iSDMS analysis, we were only able to examine the dynamic profiles of those proteins with *t*_1/2_ > 2 hr.

Protein expression is controlled by the rates of protein synthesis and degradation. Generally, higher protein synthesis rate is related to higher protein expression, and higher protein degradation rate often leads to decreased protein expression. These trends are consistent with our results (Figure 3 and Supplemental Figure 2). Traditionally, increased protein expression has been considered as being mostly attributable to increased gene transcription and translation. However, several recent global dynamic protein profiling studies have indicated that increased protein expression can also be regulated by posttranscriptional and posttranslational control [25–31]. Oksvold et al. have suggested that posttranscriptional control and increased protein half life are two of the main mechanisms to maintain protein homeostasis and buffer cells from various transcriptional and gene expression noise in response to various environmental stimulations [11].

In addition, several lines of evidence have also indicated that the abnormally elevated expression of many important receptors in cancer or drug resistant cancer cells is caused by decreased or delayed receptor degradation rather than being caused by altered gene expression [13–18]. Our results show that only ~10% of proteins with increased expression in VPS4B have decreased degradation, while the increased expression of the majority of these proteins is attributable to changes in protein synthesis only. These results strongly suggest that increasing protein synthesis or increasing translational efficiency is the main method of increasing protein expression, whereas altered protein degradation is a more protein-specific approach to increasing protein expression in SKBR3 cells. As indicated in Figure 3, we found that VPS4B-dependent protein downregulation (labeled in green diamonds) is largely caused by the combination of decreased protein synthesis and increased protein degradation, suggesting that VPS4B-mediated MVB dysfunction is playing a pivotal role in modulating protein homeostasis in breast cancer. The altered function of MVB-lysosomal degradation caused by VPS4B depletion likely stimulates other cellular degradation pathways.

VPS4B plays an important role in protein degradation, especially in the lysosomal degradation of membrane receptors [2]. It has been found that loss of VPS4B function results in delayed EGFR degradation and prolonged EGFR retention on the limiting membrane of MVBs [4–6]. However, we were not able to obtain the dynamic expression profile for EGFR and its related signaling molecules in this study due to the low abundance of endogenous EGFR and the limited dynamic proteome profile coverage in SKBR3 cells. Surprisingly, we found that overall protein degradation rates at the proteomic level were similar in SKBR3_shVPS4B and SKBR3 cells. This observation suggests that the VPS4B downregulation-induced delayed lysosomal degradation of endocytosed cargo is likely to be cargo specific and explains why overall protein degradation is not affected. Currently, we are in the process of identifying what protein cargos are specifically targeted and degraded by the Vps4B-dependent MVB-lysosomal degradation system in breast cancer. Identification of specific cargos that are delayed by VPS4B-dependent target degradation is likely going to provide critical information on whether these proteins can be used as potential biomarkers for both the classification and progression of breast cancer.

Glycolysis and fatty acid *β*-oxidation are two major metabolic pathways that cells utilize to generate energy. Adaptation to different carbon and energy sources is an important cellular response to environmental or intracellular changes. Our results indicate that dynamic protein expression of many enzymes involved in glycolysis and the TCA cycle are coordinately regulated in response to VPS4B depletion. Recently, Lin et al. have reported that there is a direct correlation between the downregulation of VPS4B and the progression of breast cancer, and that decreased VPS4B expression can be induced by hypoxia [12]. Although the molecular mechanisms underlying the hypoxia-induced VPS4B-associated tumor angiogenesis and metastasis remain unknown, our results clearly indicate that there is a direct link between altered lipid metabolism and VPS4B-mediated MVB dysfunction. 

It has been hypothesized that most cancer cells, including breast cancer cells, exhibit increased aerobic glycolysis and lead to the conversion of glucose to lactic acid, also known as the Warburg effect, in part as a result of mitochondrial respiration injury and hypoxia [41]. However, since the initial report of the Warburg hypothesis, numerous reports also indicate that many cancer cells have higher glycolytic activity even under aerobic conditions. In addition, it has been suggested by Gillies and colleagues that up-regulation of glycolysis can significantly provide many growth advantages for various cancers and the proposed conversion of glucose to lactate under aerobic conditions could be an important adaptation step for the cancer cells to survive under the intermittent hypoxic conditions during the early development of cancer (see review by Gatenby and Gillies [42]). Our current study indicated that the glycolytic pathway is downregulated in VPS4B-depleted SKBR3 cells, suggesting a potential cross-talk between the abnormal glycolysis in cancer and MVB dysfunction. Currently, we are exploring whether this attenuation of the glycolytic pathway by VPS4B could also lead to the reduction of lactate-induced acidosis that is commonly associated with many cancers. Interestingly, it has also been recently demonstrated that chronic acid-adapted MDA-MB-231 cells are able to induce the accumulation of LC3 and the possible formation of autophagosomes [43]. However, it is not currently clear whether the accumulation of LC3-positive autophagic vacuoles is due to the dysfunction of VPS4B-MVB-mediated autolysosome formation or enhanced autophagic flux. Although the main molecular and cellular events modulating this acid or environmental-induced autophagy formation in cancers are largely unknown, our systems approach described here provides a potential explanation of the role of VPS4B in regulating the glycolytic pathway and the formation of autophagy under either hypoxia or acidosis.

In addition to the downregulation of the glycolytic pathway and modulating the formation of autophagic vacuole formation, our findings also reveal that fatty acid *β*-oxidation is significantly upregulated in VPS4B-depleted cells. Specifically, we found that the expression of mitochondrial trifunctional protein alpha subunit (HADHA/ECHA), mitochondrial trifunctional protein beta subunit (HADHB/ECHB), and mitochondrial hydroxyacyl-coenzyme A dehydrogenase (HADH) is significantly elevated. The pathological consequences of abnormal fatty acid *β*-oxidation activation are unclear. However, it is reasonable to postulate that the activation of fatty acid oxidation is part of the cellular compensatory response to VPS4B-mediated downregulation of the glycolytic pathway. Fatty acid *β*-oxidation is known to be upregulated in many different cancers and other diseases [44–49]. Since mitochondria are known to be degraded by autophagy, we postulate that the VPS4B-autophagy-mediated mitochondrial degradation could be impaired during the premetastatic stage of cancer or in VPS4B-depleted cells and ultimately causes the activation of mitochondrial fatty acid *β*-oxidation. 

Finally, another important finding of our study is the decreased expression of fatty acid synthase (FASN) in VPS4B-depleted cells. High levels of FASN are reported in many epithelial cancers, such as breast, colorectal, and prostate, and FASN overexpression is highly associated with a higher risk of both disease recurrence and death [50]. Most significantly, our results show that VPS4B downregulation decreased the expression of FASN by simultaneously decreasing its rates of synthesis and increasing the rate of its degradation, suggesting that both *de novo* fatty acid synthesis and fatty acid *β*-oxidation are tightly coupled in cancer. Altogether, our study suggests the downregulation of VPS4B causes the alteration of glucose metabolism and the glycolytic pathway, which ultimately leads to the activation of mitochondrial fatty acid *β*-oxidation.

## 5. Conclusion

Here we have presented an approach, iSDMS, for the direct measurement of protein degradation and synthesis, as well as relative protein expression levels in SKBR3 cells with downregulated VPS4B expression. This approach can be feasibly applied to other cell culture systems to determine global protein dynamics under different genetic manipulations or environmental stimulations. As with other mass spectrometry-based approaches, iSDMS is limited by the depth and reproducibility of protein identification, especially when applied to multiple time points and multiple conditions. However, we are able to establish the dynamic protein profiling of many proteins involved in the central glucose and lipid metabolism that are preferentially regulated by the downregulation of VPS4B expression. As downregulation of VPS4B has been reported to be associated with certain high grade and recurring tumors, the adoption of fatty acid *β*-oxidation as an alternative energy source could be a distinct feature of breast cancer cells with VPS4B dysfunction. Therapeutic interventions targeting fatty acid ß-oxidation energy metabolism could serve as alternative and complementary strategies to treat breast cancer. 

## Supplementary Material

Supplemental Table 1: changes of protein synthesis, degradation and relative abundance induced by VPS4B down-regulation in SKBR3 cells. Supplemental Table 2: proteins with increased relative abundance at 24 hr. Supplemental Table 3: proteins with decreased relative abundance at 24 hr. Supplemental Figure 1(a-f): the dynamic protein profiles of SKBR3_shVPS4B and SKBR3 cells. Supplemental Figure 2: the relationships between protein expression and rates of protein synthesis (a) and protein degradation (b).

## Figures and Tables

**Table 1:** Increased protein expression is mostly due to increased protein synthesis in SKBR3_shVPS4B cells.

	Number of proteins*	Percentage (%)
Increased synthesis only	21	63.6
Decreased degradation only	3	9.1
Both	3	9.1
Other	6	18.2

*Number of proteins was calculated from 33 proteins with increased relative abundance (SKBR3_shVPS4B versus SKBR3 ratio >1.5) at 24 hr.

**Table 2:** Decreased protein synthesis and increased protein degradation contribute to decreased protein expression in SKBR3_shVPS4B cells.

	Number of proteins*	Percentage (%)
Decreased synthesis only	7	18.4
Increased degradation only	6	15.8
Both	25	65.8

*Number of proteins was calculated from 38 proteins with decreased relative abundance (SKBR3_shVPS4B versus SKBR3 ratio <0.66) at 24 hr.
