# Supplementary material for: An Internal Standard-Assisted Synthesis and Degradation Proteomic Approach Reveals the Potential Linkage between VPS4B Depletion and Activation of Fatty Acid β-Oxidation in Breast Cancer Cells
Source: Int J Proteomics. 2013 Feb 4;2013:291415. doi: 10.1155/2013/291415 (PMC3575666; doi:10.1155/2013/291415)
Supplement: Supplementary file 1 — Supplemental Table 1: changes of protein synthesis, degradation and relative abundance induced by VPS4B down-regulation in SKBR3 cells. Supplemental Table 2: proteins with increased relative abundance at 24 hr. Supplemental Table 3: proteins with decreased relative abundance at 24 hr. Supplemental Figure 1(a-f): the dynamic protein profiles of SKBR3_shVPS4B and SKBR3 cells. Supplemental Figure 2: the relationships between protein expression and rates of protein synthesis (a) and protein degradation (b). [file 291415.f1.pdf]

Supplementary Figure 1:

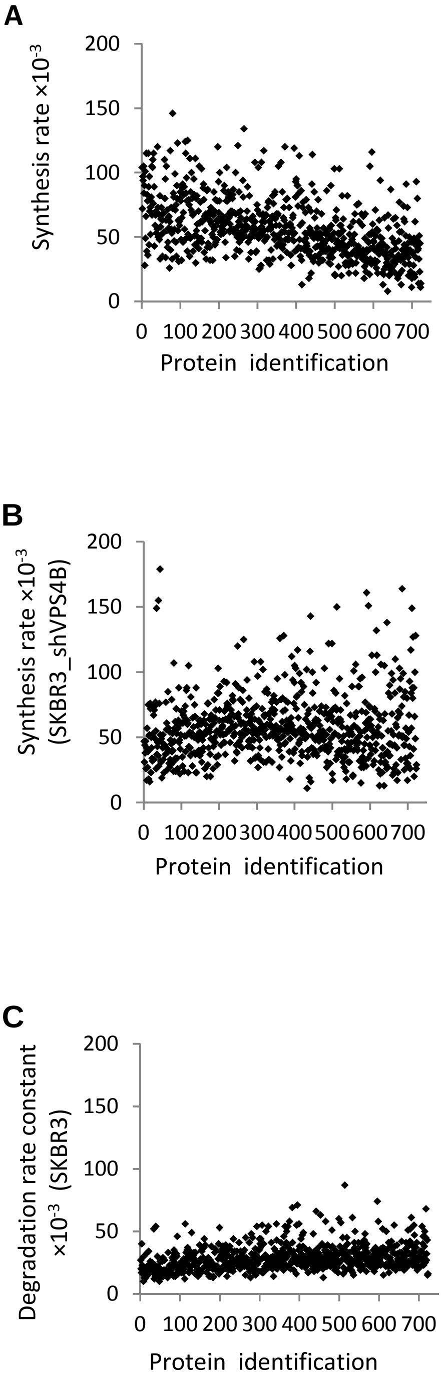

D

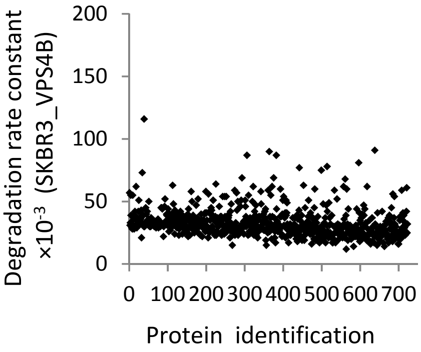

E

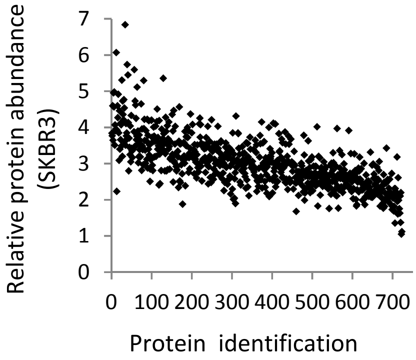

F

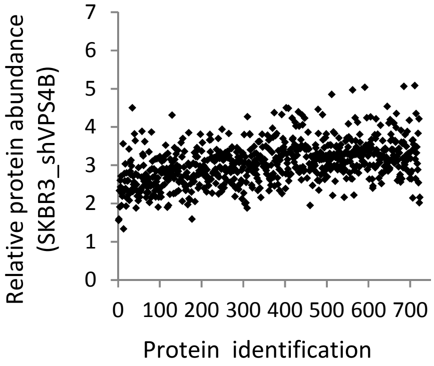

Supplementary Figure 2:

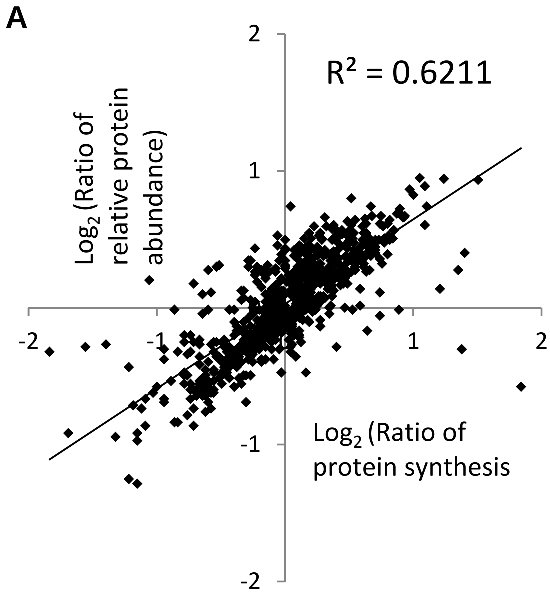

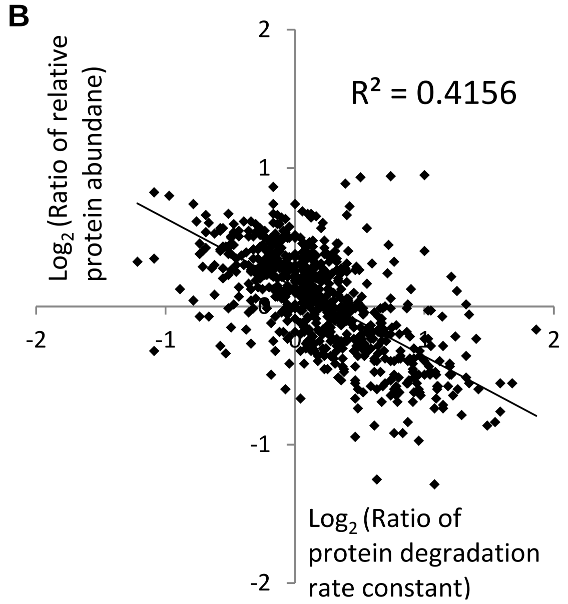

Supplemental Table 1. Changes of protein synthesis, degradation and relative abundance induced by VPS4B down-regulation in SKBR3 cells.

	**# of proteins with increased ratio* (> 1.5)**	**# of proteins with decreased ratio* (< 0.67)**	**# of proteins with unchanged ratio* (0.67 ≤ Ratio ≤ 1.5)**
Synthesis	69 (9.5%)	69 (9.5%)	585 (80.9%)
Degradation	141 (19.5%)	24 (3.3%)	558 (77.2%)
Relative protein abundance at 24 hr	33 (4.56%)	38 (5.3%)	652 (90.2%)

Numbers were calculated from a total of 723 proteins.

*: ratio = SKBR3_shVPS4B vs. SKBR3

Supplemental Table 2. Proteins with increased relative abundance at 24 hr.

Gene Name	UniProt Accession	Protein Name	Ratio* of Relative Protein Abundance at 24 hr	Ratio* of Protein Synthesis Rate	Ratio* of Protein Degradation Rate Constant
GPI	P06744	Glucose-6-phosphate isomerase	1.93	2.07	2.00
CNP	P09543	2',3'-cyclic-nucleotide 3'-phosphodiesterase	1.92	2.36	1.67
DSG2	Q14126	Desmoglein-2	1.91	2.84	1.42
PSAP	P07602	Proactivator polypeptide	1.85	2.13	1.31
ANXA2	P07355	Annexin A2	1.82	1.96	0.89
ACBD3	Q9H3P7	Golgi resident protein GCP60	1.77	2.00	0.47
HADH	Q16836	Hydroxyacyl-coenzyme A dehydrogenase, mitochondrial	1.74	1.43	0.51
CD59	P13987	CD59 glycoprotein	1.67	1.59	0.89
IDH3B	O43837	Isocitrate dehydrogenase [NAD] subunit beta, mitochondrial	1.67	1.03	0.58
KRT79	Q5XKE5	Keratin, type II cytoskeletal 79	1.67	2.15	1.00
TPM1	B7Z722	Tropomyosin 1 (Alpha), isoform CRA_i	1.65	1.86	1.34
FLNA	P21333	Filamin-A	1.61	1.83	1.04
CLTB	P09497	Clathrin light chain B	1.59	1.89	1.09
DAD1	P61803	Dolichyl-diphosphooligosaccharide--protein glycosyltransferase subunit DAD1	1.59	1.68	0.70
KRT7	P08729	Keratin, type II cytoskeletal 7	1.59	1.91	1.07
P4HA1	P13674	Prolyl 4-hydroxylase subunit alpha-1	1.59	1.60	0.89
IDH3A	P50213	Isocitrate dehydrogenase [NAD] subunit alpha, mitochondrial	1.58	1.89	0.62
LGALS3BP	Q08380	Galectin-3-binding protein	1.58	1.24	1.32
RTN4	Q9NQC3	Reticulon-4	1.58	1.90	0.91
EPB41L1	Q9H4G0	Band 4.1-like protein 1	1.57	1.54	1.12
CALD1	Q05682	Caldesmon	1.56	1.76	1.09
LMNA	P02545	Prelamin-A/C	1.56	1.46	0.93
HADHB	P55084	Trifunctional enzyme subunit beta, mitochondrial	1.55	1.40	0.95
GLB1	P16278	Beta-galactosidase	1.53	1.55	0.76
GSTK1	Q9Y2Q3	Glutathione S-transferase kappa 1	1.53	1.67	0.59
KRT14	P02533	Keratin, type I cytoskeletal 14	1.53	1.74	0.79
KHDRBS1	Q07666	KH domain-containing, RNA-binding, signal transduction-associated protein 1	1.52	1.43	0.63
RCC1	P18754	Regulator of chromosome condensation	1.52	1.30	0.89
SLC25A24	Q6NUK1	Calcium-binding mitochondrial carrier protein SCaMC-1	1.52	2.13	1.16
HADHA	P40939	Trifunctional enzyme subunit alpha, mitochondrial	1.51	1.17	0.92
CKMT1A	P12532	Creatine kinase U-type, mitochondrial	1.51	1.25	0.91
MFSD10	Q14728	Major facilitator superfamily domain-containing protein 10	1.51	1.52	0.72
RALA	P11233	Ras-related protein Ral-A	1.51	1.70	0.81

*: Ratio = SKBR3_shVPS4B vs. SKBR3

Supplemental Table 3. Proteins with decreased relative abundance at 24 hr.

Gene Name	UniProt Accession	Protein Name	Ratio* of Relative Protein Abundance at 24 hr	Ratio* of Protein Synthesis Rate	Ratio* of Protein Degradation Rate Constant
S100A9	P06702	Protein S100-A9	0.41	0.45	2.11
S100A8	P05109	Protein S100-A8	0.42	0.43	1.55
HSPB1	P04792	Heat shock protein beta-1	0.51	0.45	1.94
SLC9A3R1	O14745	Na(+)/H(+) exchange regulatory cofactor NHE-RF1	0.52	0.40	1.38
ASS1	P00966	Argininosuccinate synthase	0.53	0.31	1.78
FASN	P49327	Fatty acid synthase	0.53	0.45	1.70
GGCT	O75223	Gamma-glutamylcyclotransferase	0.55	0.61	2.80
LDHB	P07195	L-lactate dehydrogenase B chain	0.55	0.47	1.53
ARPP19	P56211	cAMP-regulated phosphoprotein 19	0.56	0.56	1.83
MIF	P14174	Macrophage migration inhibitory factor	0.56	0.55	2.92
TXN	P10599	Thioredoxin	0.58	0.59	2.44
LDHAL6A	Q6ZMR3	L-lactate dehydrogenase A-like 6A	0.59	0.65	3.00
CPNE2	Q96FN4	Copine-2	0.60	0.67	2.21
EIF5A	P63241	Eukaryotic translation initiation factor 5A-1	0.60	0.61	2.11
PHGDH	O43175	D-3-phosphoglycerate dehydrogenase	0.60	0.46	1.40
SELENBP1	Q13228	Selenium-binding protein 1	0.61	0.44	2.21
C17orf25	D3DTG9	Chromosome 17 open reading frame 25, isoform CRA_d	0.62	0.52	1.59
EEF2	P13639	Elongation factor 2	0.62	0.66	1.73
UBE2O	Q9C0C9	Ubiquitin-conjugating enzyme E2 O	0.62	0.81	1.88
ARHGDIA	P52565	Rho GDP-dissociation inhibitor 1	0.63	0.52	1.38
GART	P22102	Trifunctional purine biosynthetic protein adenosine-3	0.63	0.47	2.13
S100A13	Q99584	Protein S100-A13	0.63	0.60	1.03
HSP90AB1	P08238	Heat shock protein HSP 90-beta	0.64	0.64	1.72
PPIAL4A	Q9Y536	Peptidylprolyl cis-trans isomerase A-like 4A/B/C	0.64	0.62	1.67
S100A6	P06703	Protein S100-A6	0.64	0.65	2.33
UBE2V1	Q13404	Ubiquitin-conjugating enzyme E2 variant 1	0.64	0.65	1.96
EEF1A1	P68104	Elongation factor 1-alpha 1	0.65	0.68	1.89
IPO7	O95373	Importin-7	0.65	0.67	1.75
PDCD6	O75340	Programmed cell death protein 6	0.65	0.64	1.44
SULT1A1	P50225	Sulfotransferase 1A1	0.65	0.49	1.95
AGR2	O95994	Anterior gradient protein 2 homolog	0.66	0.62	0.95
CLIC1	O00299	Chloride intracellular channel protein 1	0.66	0.67	2.39
DNAJA1	P31689	DnaJ homolog subfamily A member 1	0.66	0.61	1.40
DDT	P30046	D-dopachrome decarboxylase	0.66	0.60	1.94
GAPDH	P04406	Glyceraldehyde-3-phosphate dehydrogenase	0.66	0.68	2.14
PPA1	Q15181	Inorganic pyrophosphatase	0.66	0.66	1.70
TUBA1B	P68363	Tubulin alpha-1B chain	0.66	0.63	2.67
UBA1	P22314	Ubiquitin-like modifier-activating enzyme 1	0.66	0.64	1.55

*: Ratio = SKBR3_shVPS4B vs. SKBR3
